# Supplementary material for: Mechanisms governing the pioneering and redistribution capabilities of the non-classical pioneer PU.1
Source: Nat Commun. 2020 Jan 21;11:402. doi: 10.1038/s41467-019-13960-2 (PMC6972792; doi:10.1038/s41467-019-13960-2)
Supplement: Supplementary file 7 — Source data [file 41467_2019_13960_MOESM7_ESM.zip › Source_Data/Figure6/Figure6B_MotifScanOutput/ETS1_mutPU.1/homerResults/motif3.similar.html]

motif3

## Information for motif3

G
T
C
A
A
G
T
C
C
G
T
A
A
G
C
T
A
C
G
T
A
C
G
T
A
G
T
C
A
G
T
C
  
Reverse Opposite:  

A
C
T
G
A
C
T
G
C
G
T
A
C
G
T
A
C
T
G
A
A
C
G
T
A
C
T
G
A
C
G
T
  

|  |  |
| --- | --- |
| p-value: | 1e-700 |
| log p-value: | -1.614e+03 |
| Information Content per bp: | 1.947 |
| Number of Target Sequences with motif | 3901.0 |
| Percentage of Target Sequences with motif | 28.93% |
| Number of Background Sequences with motif | 2807.2 |
| Percentage of Background Sequences with motif | 8.00% |
| Average Position of motif in Targets | 100.8 +/- 53.8bp |
| Average Position of motif in Background | 98.5 +/- 62.9bp |
| Strand Bias (log2 ratio + to - strand density) | 0.1 |
| Multiplicity (# of sites on avg that occur together) | 1.08 |
| Motif File: | file (matrix) reverse opposite |

### Similar de novo motifs found

|  |  |  |  |  |  |  |  |
| --- | --- | --- | --- | --- | --- | --- | --- |
| Rank | Match Score | Redundant Motif | P-value | log P-value | % of Targets | % of Background | Motif file |
| 1 | 0.759 | A C T G A C T G A C T G C T G A C G T A C T A G A C G T | 1e-61 | -140.581990 | 15.68% | 10.16% | motif file (matrix) |
